# Poly[[μ_2_-1,2-bis­(4-pyrid­yl)ethene-κ^2^ *N*:*N*′]-di-μ_3_-bromido-dicopper(I)]

**DOI:** 10.1107/S1600536810030734

**Published:** 2010-08-11

**Authors:** Fwu Ming Shen, Shie Fu Lush

**Affiliations:** a Department of Biotechnology, Yuanpei University, HsinChu 30015, Taiwan; b General Education Center, Yuanpei University, HsinChu 30015, Taiwan

## Abstract

In the title polymeric Cu^I^ compound, [Cu_2_Br_2_(C_12_H_10_N_2_)]_*n*_, the Cu cation is coordinated by an N atom from the 1,2-bis­(4-pyrid­yl)ethene ligand and three Br^−^ anions in a distorted tetra­hedral CuBr_3_N coordination geometry. Each Br^−^ anion bridges three Cu cations related by inversion centers, forming a stair-like polymeric chain along the *a* axis, and the terminal N atoms of the 1,2-bis­(4-pyrid­yl)ethene ligand, located across an inversion center, coordinate the Cu cations from neighboring chains, forming polymeric sheets.

## Related literature

For related structures, see: Yang (2009 ▶); Wang (2008 ▶); Näther & Greve (2001 ▶). For stair-like structures, see: Healy *et al.* (1989 ▶); Jasinski *et al.* (1985 ▶).
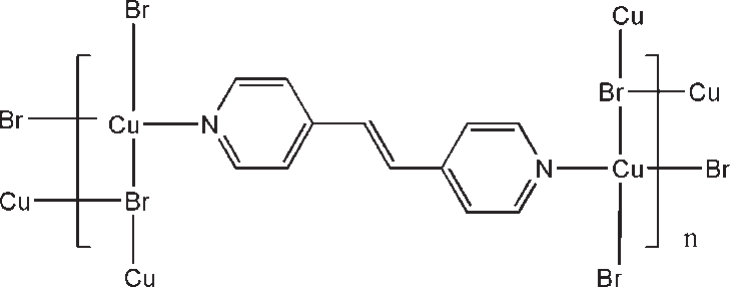


## Experimental

### 

#### Crystal data

[Cu_2_Br_2_(C_12_H_10_N_2_)]
*M* *_r_* = 234.56
Monoclinic, 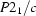

*a* = 3.9066 (3) Å
*b* = 15.1047 (13) Å
*c* = 11.1050 (9) Å
β = 95.149 (2)°
*V* = 652.64 (9) Å^3^
*Z* = 4
Mo *K*α radiation
μ = 9.36 mm^−1^
*T* = 294 K
0.40 × 0.10 × 0.05 mm

#### Data collection

Nonius KappaCCD diffractometer
Absorption correction: multi-scan (*SCALEPACK*; Otwinowski & Minor, 1997 ▶) *T*_min_ = 0.487, *T*_max_ = 0.938
3454 measured reflections
1162 independent reflections
1083 reflections with *I* > 2σ(*I*)
*R*_int_ = 0.037

#### Refinement

*R*[*F* ^2^ > 2σ(*F* ^2^)] = 0.048
*wR*(*F* ^2^) = 0.096
*S* = 1.30
1162 reflections
82 parameters
H-atom parameters constrained
Δρ_max_ = 0.61 e Å^−3^
Δρ_min_ = −0.91 e Å^−3^

### 

Data collection: *COLLECT* (Nonius, 2000 ▶); cell refinement: *SCALEPACK* (Otwinowski & Minor, 1997 ▶); data reduction: *DENZO* (Otwinowski & Minor, 1997 ▶) and *SCALEPACK*; program(s) used to solve structure: *SHELXS97* (Sheldrick, 2008 ▶); program(s) used to refine structure: *SHELXL97* (Sheldrick, 2008 ▶); molecular graphics: *PLATON* (Spek, 2009 ▶); software used to prepare material for publication: *PLATON*.

## Supplementary Material

Crystal structure: contains datablocks global, I. DOI: 10.1107/S1600536810030734/xu5009sup1.cif

Structure factors: contains datablocks I. DOI: 10.1107/S1600536810030734/xu5009Isup2.hkl

Additional supplementary materials:  crystallographic information; 3D view; checkCIF report

## Figures and Tables

**Table:** 

Br—Cu1	2.5645 (12)
Br—Cu1^i^	2.4723 (13)
Br—Cu1^ii^	2.5195 (13)
N1—Cu1	2.009 (5)

**Table:** 

Br—Cu1—N1	105.79 (16)
Br—Cu1—Br^i^	108.79 (4)
Br—Cu1—Br^ii^	110.86 (4)
Br^i^—Cu1—N1	119.11 (16)
Br^ii^—Cu1—N1	109.30 (16)
Br^i^—Cu1—Br^ii^	102.99 (4)

Symmetry codes: (i) 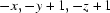; (ii) 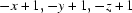.

**Fig. 1.**
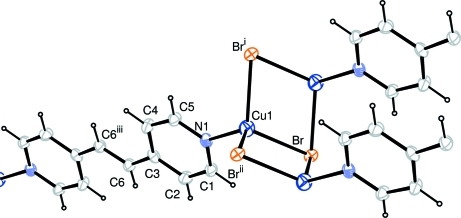
The coordination environment around the Cu(I) cation with the atom numbering. Displacement ellipsoids are drawn at the 30% probability level.

**Fig. 2.**
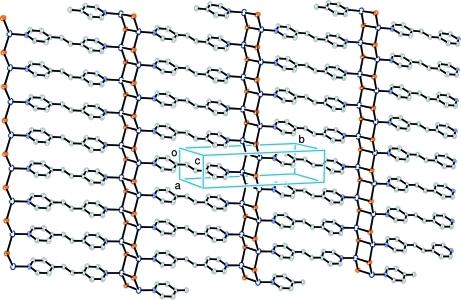
A diagram of the unit cell packing showing two-dimensional sheet structure.

## supplementary crystallographic information

### Comment

In the structural investigations of compounds of Cu^I^ halide, such as bromide (Yang, 2009; Wang, 2008; Näther & Greve, 2001), has been found. A four coordination polymer, resulted from the hydrothermal treatment of CuBr with 1,2-bis(4-pyridyl)ethene.

As Fig. 1, the symmetric unit consists of one copper(I) ion, one bromide ligand and half 1,2-bis(4-pyridyl)ethene ligand, all on general positions. The Cu^I^ atom is tetrahedral and coordinated by three µ_3_-bridging Br atoms and the each bromide bridges the other two Cu cations, while the N atoms of 1,2-bis(4-pyridyl)ethene ligand coordinate the other Cu cations, forming the three-dimensional polymeric architecture (Fig. 2).

The polymer frameworks has four-membered Cu—Br—Cu—Br units that form the step of a stair (Healy *et al.*, 1989; Jasinski *et al.*, 1985) and 1,2-bis(4-pyridyl)ethene ligand across those stairs, shown as Fig. 2. Cu···Cu distances are between 2.8852 (16)~2.9332 (16) Å and Cu—Br—Cu angles are 71.21 (4)~102.99 (4), respectively.

### Experimental

CuBr (0.1097 g, 0.50 mmol) and 1,2-bis(4-pyridyl)ethene (0.0913 g, 0.50 mmol) were mixed in 10 ml deionized water. After being stirred for 30 min, the mixture was placed in a 25 ml Teflon liner reactor and heated at 423 K in an oven for 24 h. The resulting solution was slowly cooled to room temperature. The orange transparent single crystals of the title compound were obtained in 46.45% yield.

### Refinement

H atoms were positioned geometrically with C—H = 0.93 Å, and were refined using a riding model with U_iso_(H) = 1.2U_eq_(C).

### Crystal data

**Table:**

[Cu_2_Br_2_(C_12_H_10_N_2_)]	*F*(000) = 448
*M**_r_* = 234.56	*D*_x_ = 2.387 Mg m^−^^3^
Monoclinic, *P*2_1_/*c*	Mo *K*α radiation, λ = 0.71073 Å
Hall symbol: -P 2ybc	Cell parameters from 2187 reflections
*a* = 3.9066 (3) Å	θ = 2.5–25.0°
*b* = 15.1047 (13) Å	µ = 9.36 mm^−^^1^
*c* = 11.1050 (9) Å	*T* = 294 K
β = 95.149 (2)°	Columnar, orange
*V* = 652.64 (9) Å^3^	0.40 × 0.10 × 0.05 mm
*Z* = 4	

### Data collection

**Table:**

Nonius KappaCCD diffractometer	1162 independent reflections
Radiation source: fine-focus sealed tube	1083 reflections with *I* > 2σ(*I*)
graphite	*R*_int_ = 0.037
Detector resolution: 9 pixels mm^-1^	θ_max_ = 25.1°, θ_min_ = 2.3°
CCD rotation images, thick slices scans	*h* = −4→4
Absorption correction: multi-scan (*SCALEPACK*; Otwinowski & Minor, 1997)	*k* = −17→17
*T*_min_ = 0.487, *T*_max_ = 0.938	*l* = −9→13
3454 measured reflections	

### Refinement

**Table:**

Refinement on *F*^2^	Primary atom site location: structure-invariant direct methods
Least-squares matrix: full	Secondary atom site location: difference Fourier map
*R*[*F*^2^ > 2σ(*F*^2^)] = 0.048	Hydrogen site location: inferred from neighbouring sites
*wR*(*F*^2^) = 0.096	H-atom parameters constrained
*S* = 1.30	*w* = 1/[σ^2^(*F*_o_^2^) + (0.022*P*)^2^ + 3.3443*P*] where *P* = (*F*_o_^2^ + 2*F*_c_^2^)/3
1162 reflections	(Δ/σ)_max_ = 0.010
82 parameters	Δρ_max_ = 0.61 e Å^−^^3^
0 restraints	Δρ_min_ = −0.91 e Å^−^^3^

### Special details

**Table:**

Geometry. Bond distances, angles *etc*. have been calculated using the rounded fractional coordinates. All su's are estimated from the variances of the (full) variance-covariance matrix. The cell e.s.d.'s are taken into account in the estimation of distances, angles and torsion angles
Refinement. Refinement on *F*^2^ for ALL reflections except those flagged by the user for potential systematic errors. Weighted *R*-factors *wR* and all goodnesses of fit *S* are based on *F*^2^, conventional *R*-factors *R* are based on *F*, with *F* set to zero for negative *F*^2^. The observed criterion of *F*^2^ > σ(*F*^2^) is used only for calculating -*R*-factor-obs *etc*. and is not relevant to the choice of reflections for refinement. *R*-factors based on *F*^2^ are statistically about twice as large as those based on *F*, and *R*-factors based on ALL data will be even larger.

### Fractional atomic coordinates and isotropic or equivalent isotropic displacement parameters (Å^2^)

**Table:**

	*x*	*y*	*z*	*U*_iso_*/*U*_eq_	
Br	0.19561 (17)	0.49650 (5)	0.33505 (6)	0.0347 (2)	
Cu1	0.2671 (3)	0.56165 (6)	0.54908 (9)	0.0473 (3)	
N1	0.3103 (14)	0.6931 (3)	0.5275 (5)	0.0343 (17)	
C1	0.1767 (19)	0.7337 (4)	0.4255 (6)	0.039 (2)	
C2	0.2046 (19)	0.8230 (5)	0.4055 (7)	0.041 (2)	
C3	0.3818 (18)	0.8766 (4)	0.4912 (6)	0.034 (2)	
C4	0.520 (2)	0.8355 (5)	0.5972 (6)	0.040 (2)	
C5	0.4799 (18)	0.7450 (4)	0.6095 (6)	0.037 (2)	
C6	0.4128 (19)	0.9711 (4)	0.4647 (7)	0.036 (2)	
H1	0.05970	0.69940	0.36560	0.0470*	
H2	0.10420	0.84770	0.33410	0.0490*	
H4	0.63640	0.86840	0.65860	0.0480*	
H5	0.57850	0.71860	0.67990	0.0440*	
H6	0.30120	0.99200	0.39280	0.0440*	

### Atomic displacement parameters (Å^2^)

**Table:**

	*U*^11^	*U*^22^	*U*^33^	*U*^12^	*U*^13^	*U*^23^
Br	0.0345 (4)	0.0376 (4)	0.0312 (4)	−0.0036 (3)	−0.0006 (3)	0.0001 (3)
Cu1	0.0589 (6)	0.0302 (5)	0.0520 (6)	−0.0054 (4)	0.0000 (4)	0.0062 (4)
N1	0.041 (3)	0.023 (3)	0.039 (3)	−0.001 (2)	0.005 (3)	0.000 (2)
C1	0.048 (4)	0.029 (4)	0.038 (4)	0.002 (3)	−0.006 (3)	−0.002 (3)
C2	0.049 (4)	0.036 (4)	0.035 (4)	0.002 (3)	−0.006 (3)	0.007 (3)
C3	0.038 (4)	0.023 (3)	0.041 (4)	0.003 (3)	0.011 (3)	0.006 (3)
C4	0.050 (4)	0.035 (4)	0.035 (4)	−0.006 (3)	−0.001 (3)	−0.006 (3)
C5	0.043 (4)	0.029 (3)	0.037 (4)	−0.004 (3)	−0.003 (3)	0.001 (3)
C6	0.043 (4)	0.029 (4)	0.036 (4)	0.002 (3)	−0.001 (3)	0.000 (3)

### Geometric parameters (Å, °)

**Table:**

Br—Cu1	2.5645 (12)	C3—C6	1.465 (9)
Br—Cu1^i^	2.4723 (13)	C4—C5	1.384 (10)
Br—Cu1^ii^	2.5195 (13)	C6—C6^iii^	1.321 (10)
N1—Cu1	2.009 (5)	C1—H1	0.9300
N1—C1	1.351 (8)	C2—H2	0.9300
N1—C5	1.332 (8)	C4—H4	0.9300
C1—C2	1.373 (10)	C5—H5	0.9300
C2—C3	1.386 (10)	C6—H6	0.9300
C3—C4	1.396 (10)		
			
Br···C1	3.724 (6)	C4···H6^iii^	2.7000
Br···H1	3.1300	C6···H4^iii^	2.7800
Br···H2^iv^	3.0900	H1···Br	3.1300
Br···H6^iv^	3.0500	H2···H6	2.3800
C1···C5^v^	3.551 (10)	H2···Br^ix^	3.0900
C2···C3^v^	3.527 (10)	H4···C6^iii^	2.7800
C2···C4^v^	3.570 (11)	H4···H6^iii^	2.2000
C3···C2^vi^	3.527 (10)	H5···C2^x^	3.0800
C4···C2^vi^	3.570 (11)	H6···H2	2.3800
C5···C1^vi^	3.551 (10)	H6···Br^ix^	3.0500
C6···C6^vii^	3.498 (10)	H6···C4^iii^	2.7000
C2···H5^viii^	3.0800	H6···H4^iii^	2.2000
			
Cu1—Br—Cu1^i^	71.21 (4)	C2—C3—C6	118.5 (6)
Cu1—Br—Cu1^ii^	69.15 (4)	C4—C3—C6	124.8 (6)
Cu1^i^—Br—Cu1^ii^	102.99 (4)	C3—C4—C5	118.9 (6)
Br—Cu1—N1	105.79 (16)	N1—C5—C4	124.6 (6)
Br—Cu1—Br^i^	108.79 (4)	C3—C6—C6^iii^	124.9 (7)
Br—Cu1—Br^ii^	110.86 (4)	N1—C1—H1	118.00
Br^i^—Cu1—N1	119.11 (16)	C2—C1—H1	118.00
Br^ii^—Cu1—N1	109.30 (16)	C1—C2—H2	120.00
Br^i^—Cu1—Br^ii^	102.99 (4)	C3—C2—H2	120.00
Cu1—N1—C1	121.2 (4)	C3—C4—H4	121.00
Cu1—N1—C5	122.8 (4)	C5—C4—H4	121.00
C1—N1—C5	115.9 (5)	N1—C5—H5	118.00
N1—C1—C2	123.5 (6)	C4—C5—H5	118.00
C1—C2—C3	120.3 (7)	C3—C6—H6	118.00
C2—C3—C4	116.8 (6)	C6^iii^—C6—H6	118.00
			
Cu1^i^—Br—Cu1—N1	−129.06 (17)	Br^i^—Cu1—N1—C1	−97.7 (5)
Cu1^ii^—Br—Cu1—N1	118.37 (17)	Cu1—N1—C1—C2	−178.8 (6)
Cu1^i^—Br—Cu1—Br^i^	0.00 (4)	C5—N1—C1—C2	−1.0 (10)
Cu1^ii^—Br—Cu1—Br^i^	−112.57 (5)	Cu1—N1—C5—C4	179.0 (6)
Cu1^i^—Br—Cu1—Br^ii^	112.57 (5)	C1—N1—C5—C4	1.2 (10)
Cu1^ii^—Br—Cu1—Br^ii^	0.00 (4)	N1—C1—C2—C3	1.1 (11)
Cu1^i^—Br^i^—Cu1—N1	121.22 (19)	C1—C2—C3—C4	−1.3 (11)
Cu1^ii^—Br^ii^—Cu1—Br	0.00 (4)	C1—C2—C3—C6	178.4 (7)
Cu1^ii^—Br^ii^—Cu1—N1	−116.22 (17)	C2—C3—C4—C5	1.4 (10)
Cu1^i^—Br^i^—Cu1—Br	0.00 (5)	C6—C3—C4—C5	−178.3 (7)
Br^ii^—Cu1—N1—C1	144.4 (5)	C2—C3—C6—C6^iii^	−176.7 (7)
Br^ii^—Cu1—N1—C5	−33.2 (6)	C4—C3—C6—C6^iii^	3.0 (12)
Br^i^—Cu1—N1—C5	84.7 (5)	C3—C4—C5—N1	−1.5 (11)
Br—Cu1—N1—C1	25.0 (5)	C3—C6—C6^iii^—C3^iii^	−180.0 (7)
Br—Cu1—N1—C5	−152.6 (5)		

Symmetry codes: (i) −*x*, −*y*+1, −*z*+1; (ii) −*x*+1, −*y*+1, −*z*+1; (iii) −*x*+1, −*y*+2, −*z*+1; (iv) −*x*, *y*−1/2, −*z*+1/2; (v) *x*−1, *y*, *z*; (vi) *x*+1, *y*, *z*; (vii) −*x*, −*y*+2, −*z*+1; (viii) *x*, −*y*+3/2, *z*−1/2; (ix) −*x*, *y*+1/2, −*z*+1/2; (x) *x*, −*y*+3/2, *z*+1/2.
